# Supplementary figures and images for: Phage-induced efflux down-regulation boosts antibiotic efficacy
Source: PLoS Pathog. 2024 Jun 28;20(6):e1012361. doi: 10.1371/journal.ppat.1012361 (PMC11239113; doi:10.1371/journal.ppat.1012361)

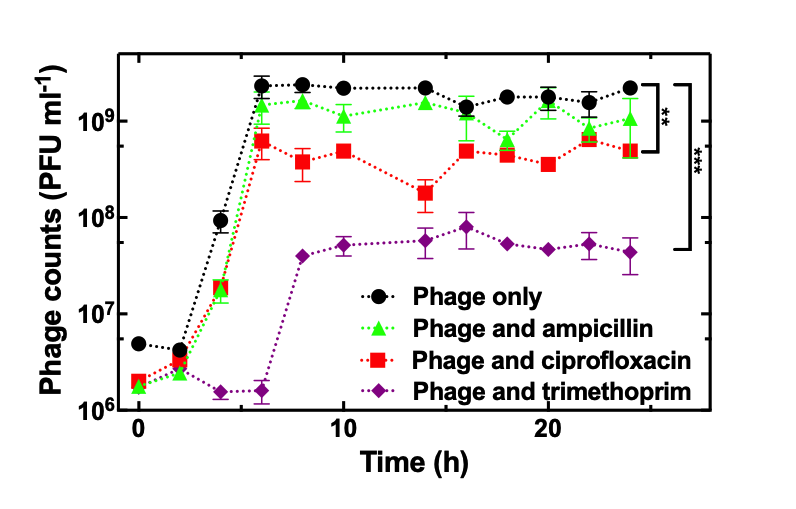

Supplement: S1 Fig — Temporal dependence of phage counts from stationary phase B. thailandensis cultures incubated in LB medium only (black circles), or in LB medium containing ampicillin (green triangles), ciprofloxacin (red squares) or trimethoprim (purple diamonds) at 0.25× their respective MIC values. In all cases the initial bacterial inoculum was 2×10^6^ CFU ml-1 and the starting concentration of phage was 2×10^6^ PFU ml-1. Symbols and error bars are means and standard errors of the mean of phage count measurements from biological and technical triplicates. Very small error bars cannot be visualised due to overlap with the datapoints. Dashed lines are guides-for-the-eye. **** indicate a p-value < 0.0001. Numerical values are reported in S2 File. (TIFF) [file ppat.1012361.s001.tiff]

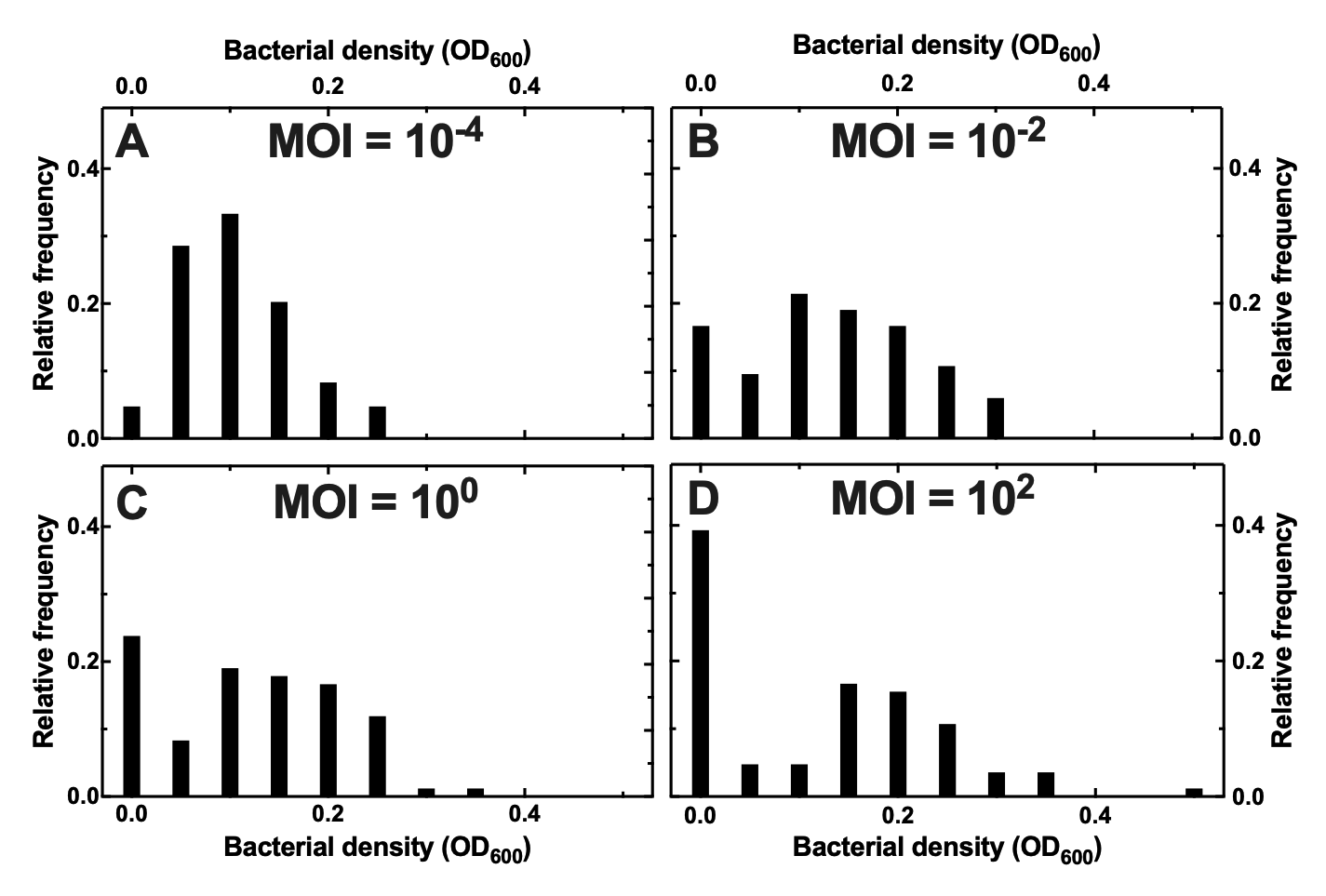

Supplement: S2 Fig — Distribution of B. thailandensis density values, measured in OD600, after 24 h exposure to phage at an MOI of (A) 10−4, (B) 10−2, (C) 100 or (D) 102. For each condition, bacterial density measurements were carried out in 84 independent micro-cultures from biological triplicates. Numerical values are reported in Data C in S1 File. (TIFF) [file ppat.1012361.s002.tiff]

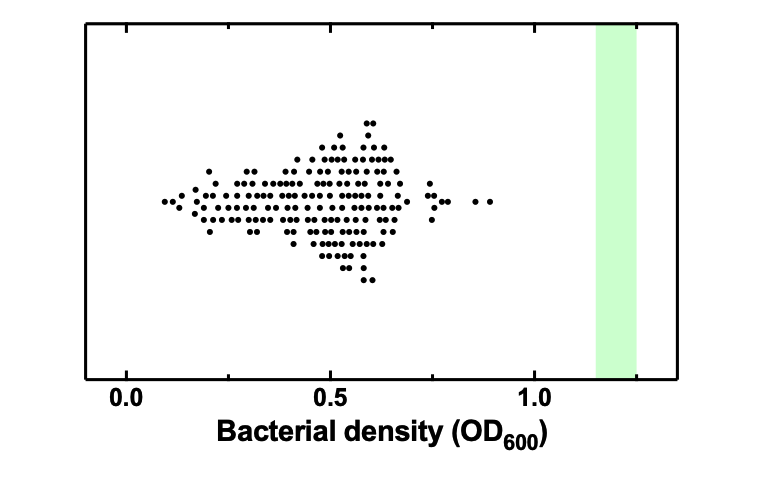

Supplement: S3 Fig — Bacterial density measurements after 72 h incubation at 25°C in the presence of phage at an MOI of 1. Each black circle represents a bacterial density value performed on one of 84 technical micro-culture replicates from biological triplicates. The green vertical band represents the mean and standard error of the mean of corresponding bacterial density measurements in the absence of phage. Numerical values are reported in Data C in S1 File. (TIFF) [file ppat.1012361.s003.tiff]

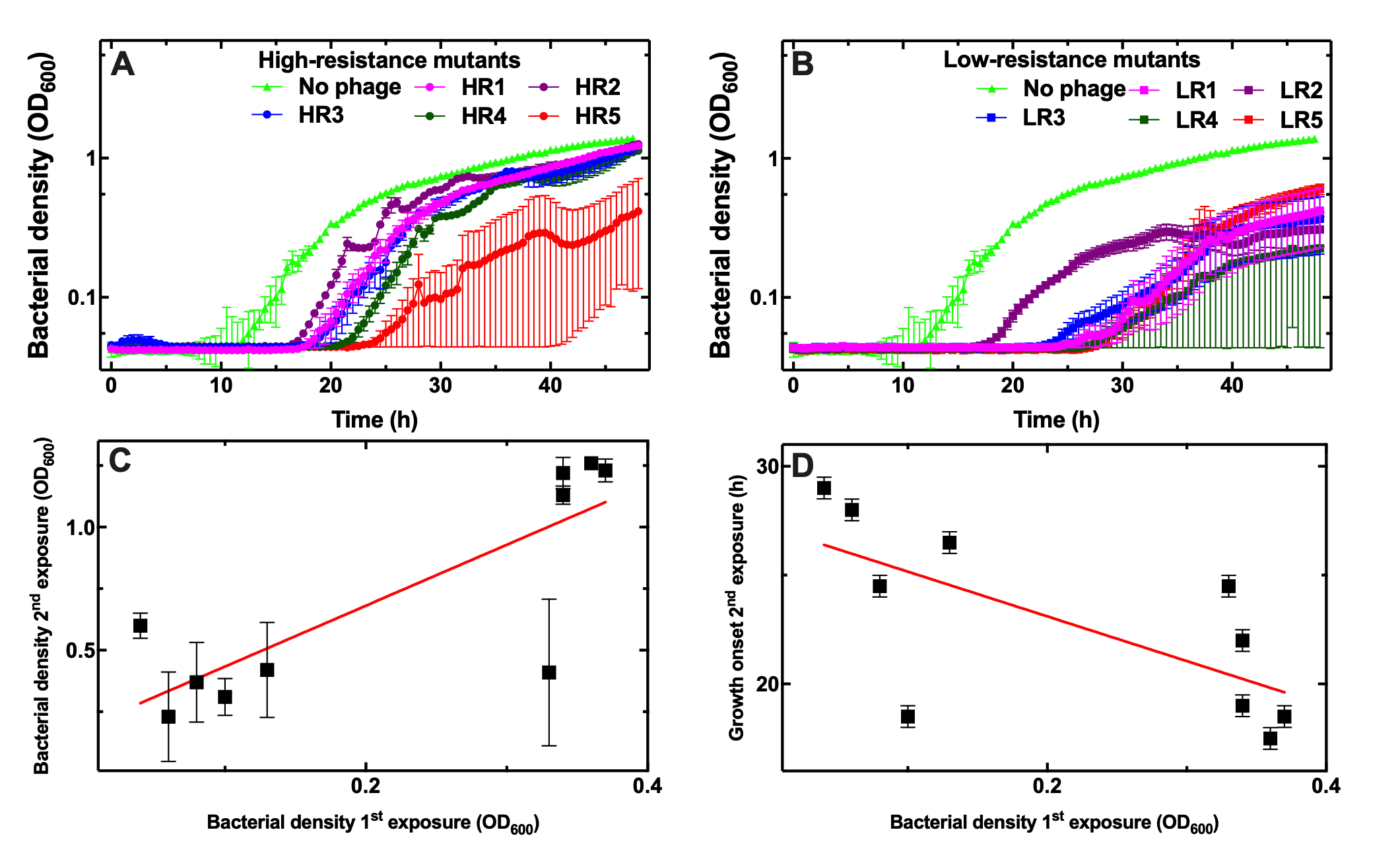

Supplement: S4 Fig — Growth of (A) high-resistant and (B) low-resistant mutants when re-inoculated in 96-well plates in the presence of phage at an MOI of 1 for 48h. Data points for each mutant have been color coded as in Fig 1C and 1D, HR and LR stands for high-resistant and low-resistant, respectively, and mutants have been numbered in descending order of resistance according to the data in Fig 1C, i.e. HR1 is the highest resistant mutant whereas LR5 is the lowest resistant mutant investigated. Green triangles represent the growth of the parental strain in the absence of phage. Symbols and error bars are means and standard errors of bacterial density values, measured in OD600, obtained from 9 technical replicates from biological triplicates. Corresponding correlation between (C) the final optical density at the end of the second 48h exposure to phage and the optical density measured for each survivor population at the end of the first 48h exposure to phage and (D) the onset of growth during the second exposure to phage and the optical density measured for each survivor population at the end of the first 48h exposure to phage. The red lines are linear regressions to the data. Numerical values are reported in S3 File. (TIFF) [file ppat.1012361.s004.tiff]

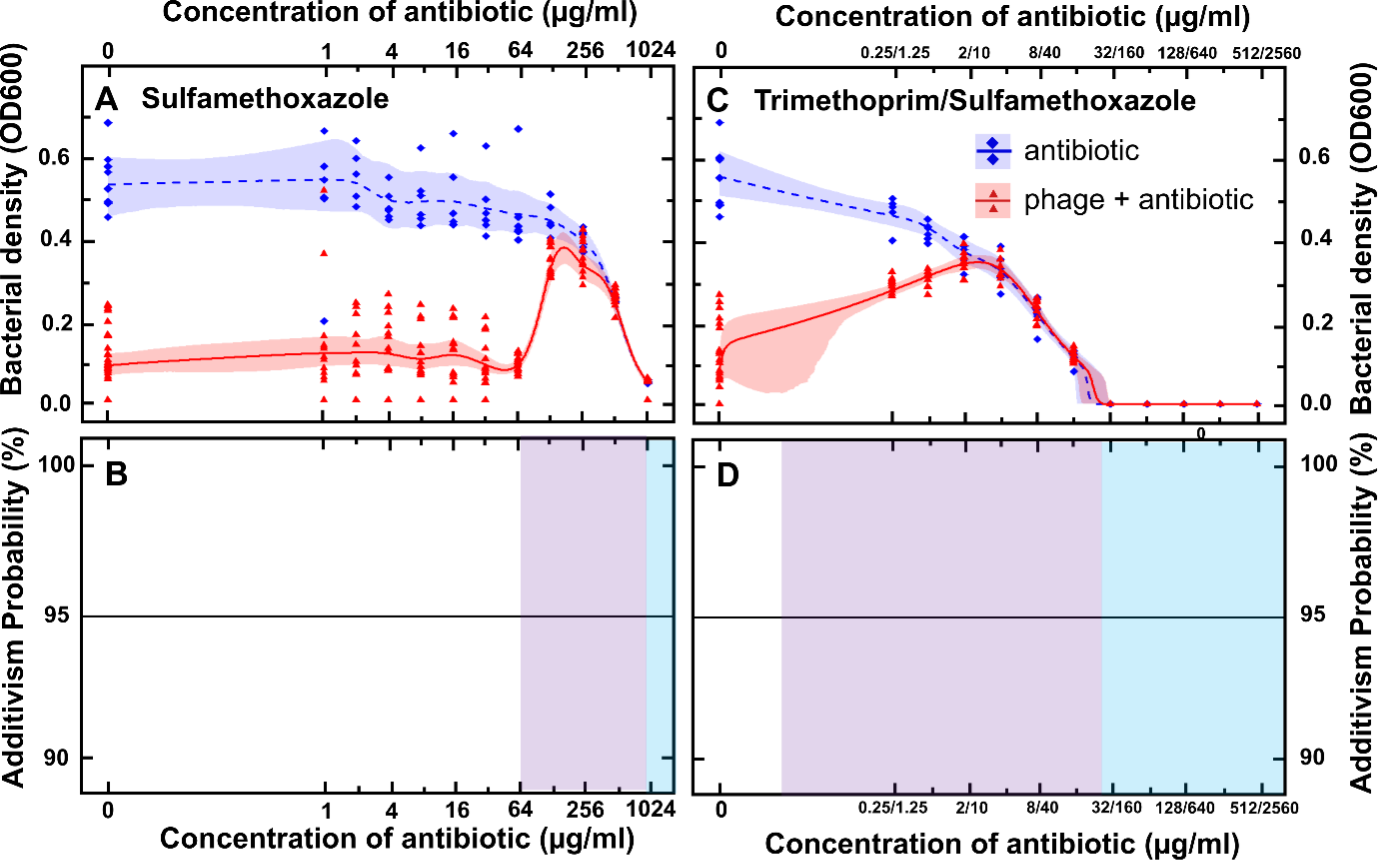

Supplement: S5 Fig — Experimental data (symbols) and model predictions (lines and bands) describing the dependence of bacterial density on the concentration of (A) sulfamethoxazole and (C) trimethoprim/sulfamethoxazole (at a ratio of 1:5) in the absence (blue diamonds) and presence of phage ΦBp-AMP1 at an MOI of 1 (red triangles) after 24 h treatment. Each symbol represents the bacterial density measured in one of 15 technical replicates collated from biological triplicates. Some of the symbols overlap with each other. The lines and shaded areas are the medians, upper and lower quartiles, estimated by fitting our statistical non-linear regression model to our experimental data via Markov Chain Monte Carlo simulations. Corresponding predicted probability of an additive interaction between phage and (B) sulfamethoxazole and (D) trimethoprim/ sulfamethoxazole (ratio 1:5) is below 90%. Not shaded or blue shaded areas indicate antibiotic concentration ranges where the phage or the antibiotic dominate, respectively. Purple shaded areas indicate antagonism. Numerical values are reported in Data B in S5 File. (TIF) [file ppat.1012361.s005.tif]

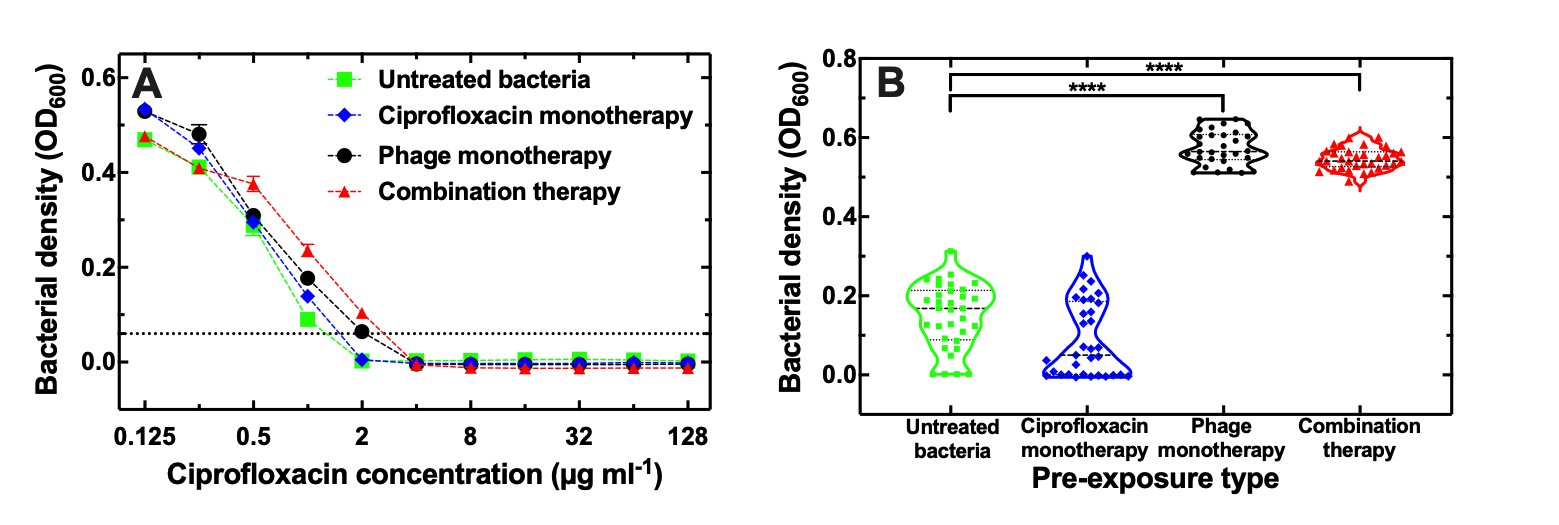

Supplement: S6 Fig — (A) Dependence of bacterial density, measured in OD600, on the concentration of ciprofloxacin for B. thailandensis that had not undergone any previous therapy (green squares), B. thailandensis that had undergone a 24 h monotherapy with ciprofloxacin at 0.125× its MIC (blue diamonds), B. thailandensis that had undergone a 24 h monotherapy with phage at an initial MOI of 1 (black circles), or B. thailandensis that had undergone a 24 h combination therapy with ciprofloxacin at 0.125× its MIC and phage at an initial MOI of 1 (red triangles). Symbols and error bars are means and standard errors of the means of bacterial density measurements obtained from biological triplicates each containing five technical micro-culture replicates. Very small error bars cannot be visualised due to overlap with the datapoints. Dashed lines are guides-for-the-eye. The horizontal dashed line represents 10% of the bacterial density value measured after 24 h incubation in LB medium in the absence of ciprofloxacin and phage. (B) Bacterial density, measured in OD600, after 24 h exposure to phage at an initial MOI of 1 for B. thailandensis that had not undergone any previous therapy (green squares), B. thailandensis that had undergone a 24 h monotherapy with ciprofloxacin at 0.125× its MIC (blue diamonds), B. thailandensis that had undergone a 24 h monotherapy with phage at an initial MOI of 1 (black circles), or B. thailandensis that had undergone a 24 h combination therapy with ciprofloxacin at 0.125× its MIC and phage at an initial MOI of 1 (red triangles). Each symbol represents a bacterial density measurement obtained from one of 30 technical micro-culture replicates from biological triplicates. The median and quartile of each distribution are indicated as black dashed and dotted lines, respectively. Numerical values are reported in Data C and D in S7 File. (TIFF) [file ppat.1012361.s006.tiff]

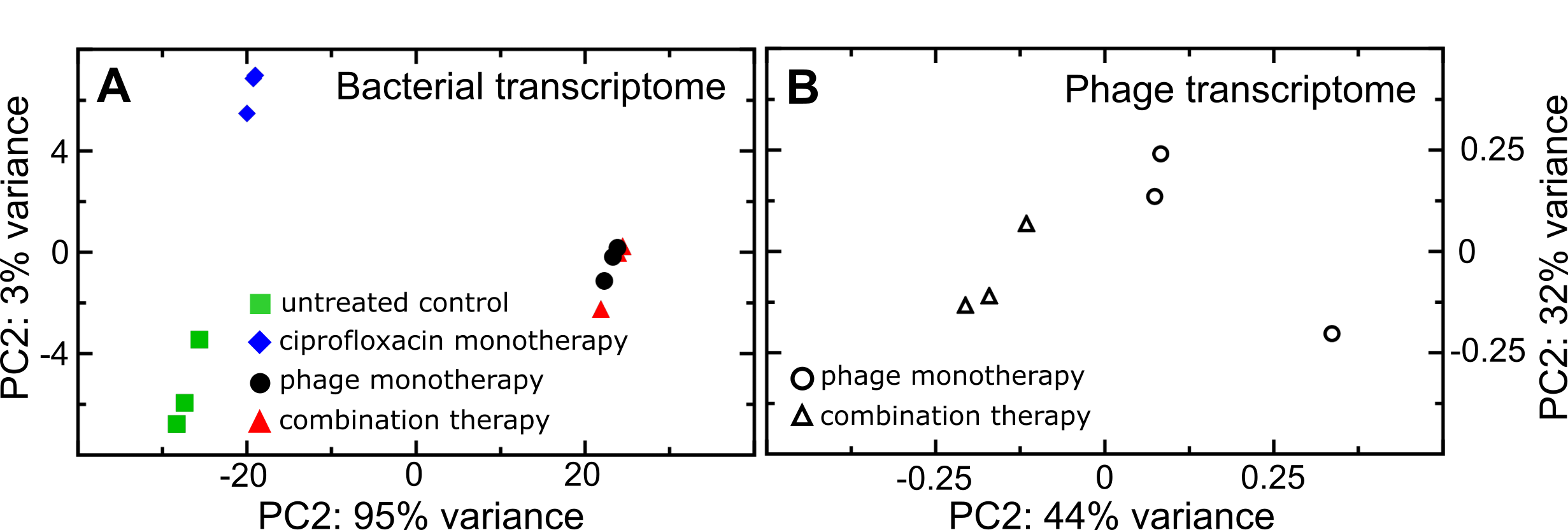

Supplement: S7 Fig — (A) Principal component analysis of replicate transcriptomes of stationary phase B. thailandensis incubated for 4 h either in LB medium only (green squares), or in LB medium containing ciprofloxacin at 0.125× its MIC (blue diamonds), or LB medium containing phage at an MOI of 1 (black circles), or in LB medium containing both ciprofloxacin at 0.125× its MIC and phage at an MOI of 1 (red triangles). (B) Corresponding principal component analysis of replicate transcriptomes of phage ΦBp-AMP1 after 4 h incubation with stationary phase B. thailandensis in LB medium only (open circles) or in LB medium containing ciprofloxacin at 0.125× its MIC (open triangles). Note the narrower x-axis range with respect to S6A Fig because of the largely overlapping phage transcriptomes. Numerical values are reported in S11 File. (TIF) [file ppat.1012361.s007.tif]

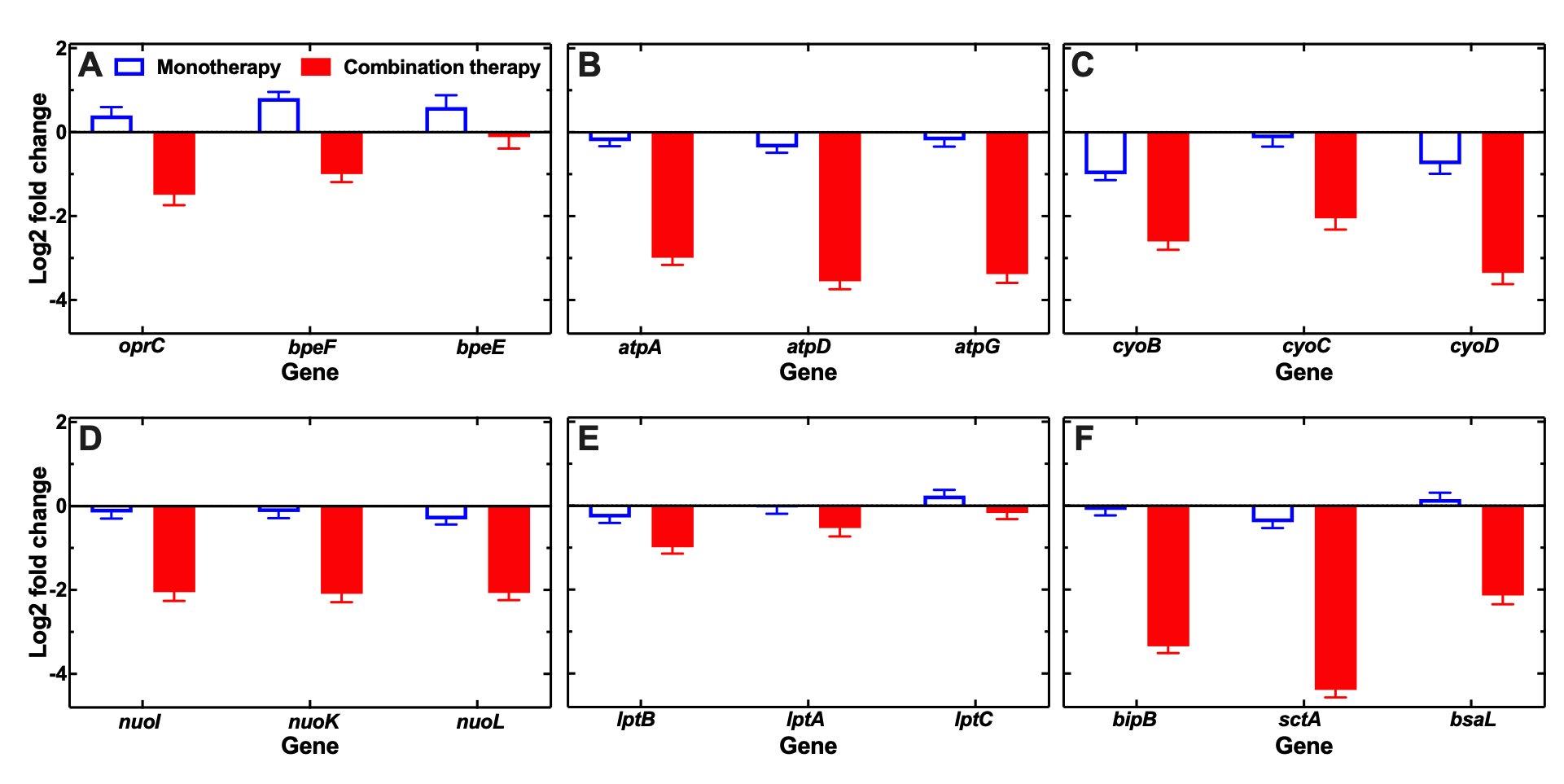

Supplement: S8 Fig — Downregulated (negative Log2-fold change) or upregulated (positive Log2-fold change) genes of interest in stationary phase B. thailandensis after 4 h of monotherapy with ciprofloxacin at 0.125× MIC (blue empty bars) or combination therapy with ciprofloxacin at 0.125× MIC and phage at an MOI of 1 (red filled bars) with respect to untreated B. thailandensis incubated for 4 h in LB medium only. Corresponding numerical values are reported in S8 and S9 File. (TIFF) [file ppat.1012361.s008.tiff]

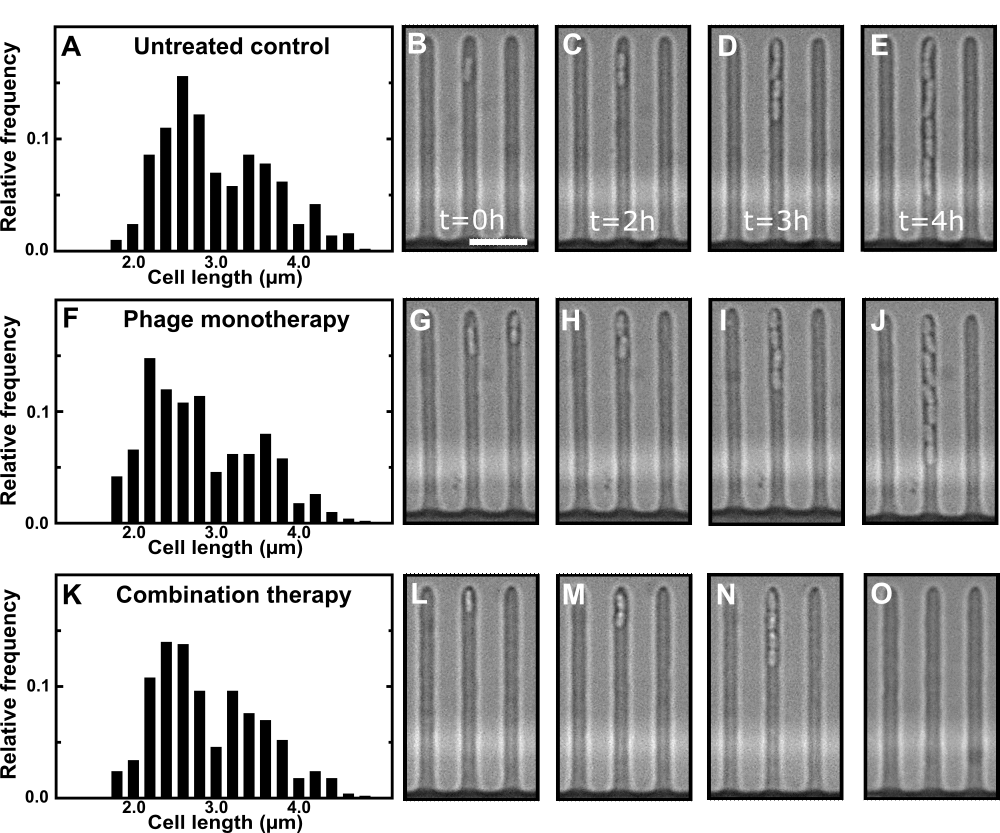

Supplement: S9 Fig — Distribution of single-cell lengths for (A) B. thailandensis incubated in LB medium only, (F) B. thailandensis incubated in LB medium containing phage at a concentration of 2×10^8^ PFU ml-1 and (K) B. thailandensis incubated in LB medium containing both phage at a concentration of 2×10^8^ PFU ml-1 and ciprofloxacin at 0.125× its MIC. Each distribution contains 500 single-cell length measurements carried out on bacteria hosted in different microfluidic compartments from different biological triplicate experiments over a period of 9 h exposure to each condition. Corresponding representative microscopy images are reported in (B-E), (G-J) and (L-O), respectively. Scale bar: 5 μm. Numerical values are reported in S17 File. (TIF) [file ppat.1012361.s009.tif]

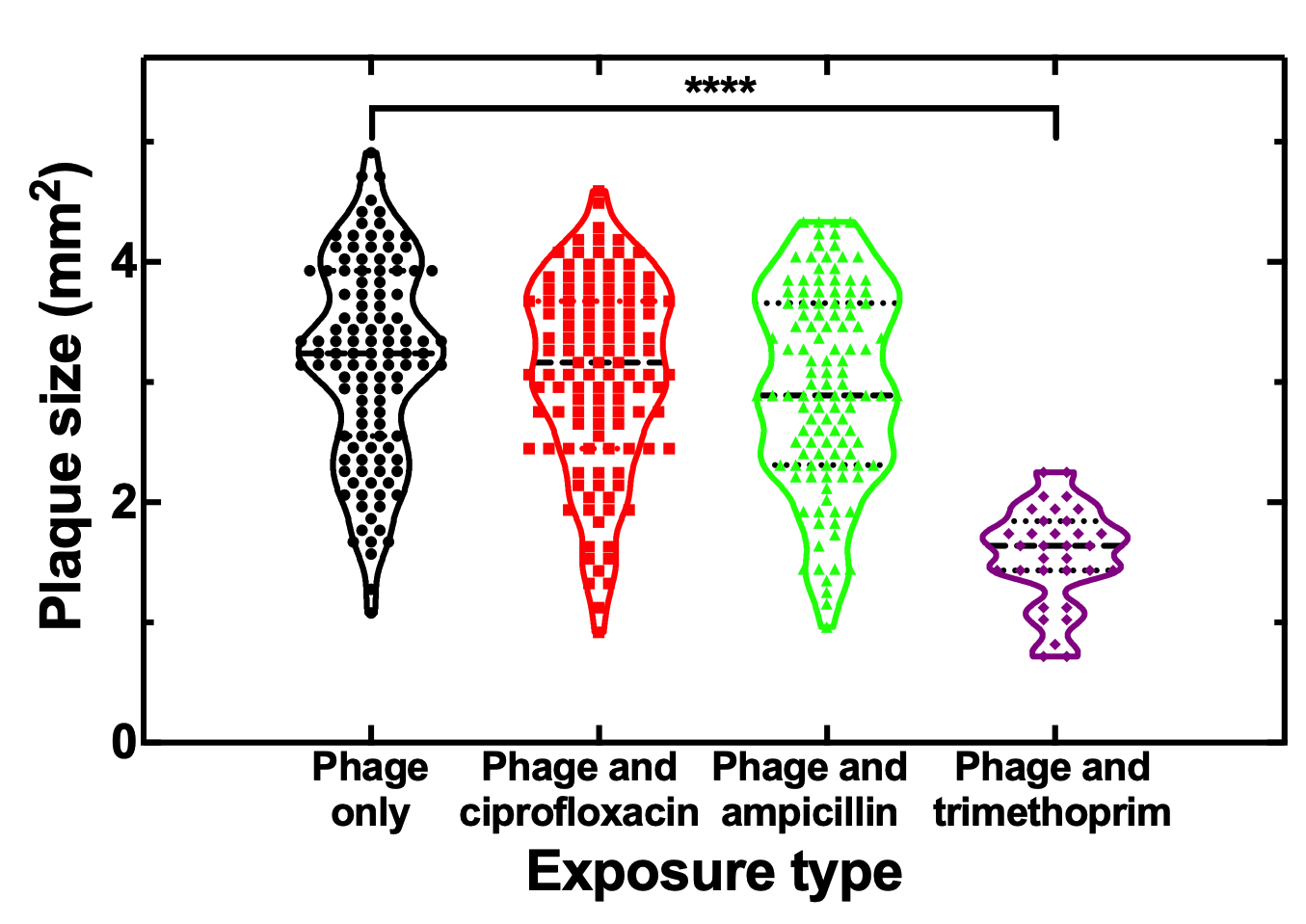

Supplement: S10 Fig — Distribution of sizes of plaques formed ΦBp-AMP1 when plated on B. thailandensis alone (black circles), or in the presence of ciprofloxacin (red squares), ampicillin (green triangles) or trimethoprim (purple diamonds) at 0.125× their respective MIC values. Each symbol reports a plaque size value measured on one of 150 plaques for each condition. Dashed and dotted horizontal lines represent the median and quartiles, respectively, of each distribution. **** indicates a p-value < 0.0001. Similar data were obtained when these antibiotics were used at 0.5× their respective MIC values. Numerical values are reported in S18 File. (TIFF) [file ppat.1012361.s010.tiff]
