# Supplementary material for: Phage-induced efflux down-regulation boosts antibiotic efficacy
Source: PLoS Pathog. 2024 Jun 28;20(6):e1012361. doi: 10.1371/journal.ppat.1012361 (PMC11239113; doi:10.1371/journal.ppat.1012361)
Supplement: S1 Table — Mutant name, gene, gene product, annotation and unique mutation measured in the five high- and low-resistant populations indicated in Fig 1C and 1D. (DOCX) [file ppat.1012361.s011.docx]

| **Mutant name** | **Gene** | **Product** | **Annotation** | **Mutation** |
| --- | --- | --- | --- | --- |
| HR1 | *BTH_RS19765 →* | Polysaccharide biosynthesis protein | H478Y (CAT→TAT) | C→T |
| HR2 | *BTH_RS19745 →* | Glycosyltransferase | I231S (ATC→AGC) | T→G |
| HR4 | *BTH_RS19745 →* | Glycosyltransferase | P521L (CCG→CTG) | C→T |
| HR5 | *BTH_RS19740 →* | O-antigen methyl transferase | S476P (TCC→CCC) | T→C |
| LR1 | *BTH_RS14320 →* | O‑acetyl‑ADP‑ribose deacetylase | V38G (GTC→GGC) | T→G |
| LR1 | *wbiB →* | dTDP‑L‑rhamnose 4‑epimerase | coding (415/1092 nt) | (CCGAGCAG)_1→2_ |
| LR2 | *mlaE ←* | ABC transporter subunit MlaE | *256Y (TAA→TAC) | T→G |
| LR4 | *rfbA →* | Thymidylyltransferase | coding (675/894 nt) | Δ1 bp |
| LR5 | *wbiB →* | dTDP‑L‑rhamnose 4‑epimerase | coding (473/1092 nt) | +G |
| LR5 | *BTH_RS21230 →/-35* | Hypothetical lipoprotein | intergenic (+131/‑129) | (AAGGGCTC)_7→8_ |
