# Supplementary material for: Phage-induced efflux down-regulation boosts antibiotic efficacy
Source: PLoS Pathog. 2024 Jun 28;20(6):e1012361. doi: 10.1371/journal.ppat.1012361 (PMC11239113; doi:10.1371/journal.ppat.1012361)
Supplement: S2 Table — Score of integrity for Bulkholderia phage ΦBp-AMP1, ΦE12-2, ΦE125, KL3 and vB_BmuP_KL4, and the Ralstonia phage RsoM1USA measured from 5 representative high-resistant mutant cultures, 5 representative low-resistant mutant cultures and 5 untreated control cultures, all in the absence of externally added phage. Detected phage sequences were given a score of integrity via PHASTER, with a maximum value of 150, scores above 90 were considered as intact prophages (blue shaded tabs), scores below 70 were considered as incomplete (red shaded tabs). (DOCX) [file ppat.1012361.s012.docx]

| **Level of resistance** | ***Burkholderia phage*** | | | | | ***Ralstonia phage*** |
| --- | --- | --- | --- | --- | --- | --- |
|  | **ΦBp-AMP1** | **ΦE12-2** | **ΦE125** | **KL3** | **vB_BmuP_KL4** | **RsoM1USA** |
|  | NC_047743 | NC_009236 | NC_003309 | NC_015266 | NC_047958 | NC_049432 |
| **High** | 0 | 150 | 150 | 20 | 0 | 0 |
|  | 0 | 150 | 150 | 0 | 0 | 20 |
|  | 0 | 150 | 150 | 0 | 0 | 20 |
|  | 0 | 150 | 150 | 40 | 0 | 20 |
|  | 0 | 150 | 150 | 40 | 0 | 20 |
| **Low** | 0 | 150 | 140 | 40 | 0 | 20 |
|  | 0 | 150 | 150 | 0 | 0 | 20 |
|  | 0 | 150 | 150 | 0 | 0 | 20 |
|  | 0 | 150 | 150 | 0 | 40 | 20 |
|  | 0 | 150 | 150 | 0 | 0 | 20 |
| **Untreated Control** | 0 | 150 | 150 | 0 | 0 | 20 |
|  | 0 | 150 | 150 | 0 | 40 | 20 |
|  | 0 | 150 | 150 | 40 | 0 | 20 |
|  | 0 | 150 | 150 | 40 | 0 | 20 |
|  | 0 | 150 | 150 | 40 | 0 | 20 |
