# Supplementary material for: Phage-induced efflux down-regulation boosts antibiotic efficacy
Source: PLoS Pathog. 2024 Jun 28;20(6):e1012361. doi: 10.1371/journal.ppat.1012361 (PMC11239113; doi:10.1371/journal.ppat.1012361)
Supplement: S3 Table — Antibiotic class, antibiotic molecule and range of antibiotic concentrations for which our model predicted additivism between phage and each antibiotic based on our experimental MIC values recorded for monotherapy and combination therapy using each antibiotic. Interactions between phage and antibiotics were considered to be additive if the probability that combination therapy was more effective in inhibiting bacterial growth than phage and antibiotic monotherapies was above 95%. (DOCX) [file ppat.1012361.s013.docx]

| **Class** | **Molecule** | **Predicted  additive range (µg ml^-1^)** |
| --- | --- | --- |
| Quinolones | Nalidixic Acid | 2 - 16 |
|  | Ciprofloxacin | 0.06 - 2 |
|  | Ofloxacin | 0.5 - 4 |
|  | Levofloxacin | 0.5 - 4 |
|  | Finafloxacin | 0.06 - 2 |
|  | Moxifloxacin | 0.06 - 2 |
| β-Lactams | Amoxicillin | 12 - 32 |
|  | Ampicillin | 12 - 32 |
|  | Cefaclor | 64 - 256 |
|  | Ceftazidime | 0.5 - 2.5 |
|  | Meropenem | 1.25 - 4 |
| Tetracycline | Doxycycline | 1 - 4 |
|  | Tetracycline | 2 - 8 |
