# Supplementary material for: Phage-induced efflux down-regulation boosts antibiotic efficacy
Source: PLoS Pathog. 2024 Jun 28;20(6):e1012361. doi: 10.1371/journal.ppat.1012361 (PMC11239113; doi:10.1371/journal.ppat.1012361)
Supplement: Attachment — Submitted filename: PLoS Pathogens Kraus et al_Response to reviewers.pdf [file ppat.1012361.s032.pdf]

Major issues, Reviewer 2

The proposed mechanism for cipro-phage synergy is not further tested by showing a dependence of "phage synergy" on the presence of the efflux system (i.e. examining mutant strain), directly measuring bacterial cell membrane potential ± phage infection (using a phage-R mutant as a control) or by showing a generalized effect of phage infection on efflux of other substrates (eg Nile Red).

Following the Reviewer's suggestion, we have now performed new experiments measuring and contrasting the accumulation of Syto 9, a substrate of efflux pumps (Minero et al. Analyst, 10.1039/d3an02112b 2024), in the presence and absence of ΦBp-AMP1. In accordance with our ciprofloxacin-NBD data, we found that the distribution of Syto 9 fluorescence values after 1 h of supply in the presence of phage was significantly higher than the distribution of Syto 9 fluorescence values after 1 h of supply in the absence of phage (p-value < 0.0001, Figure 6D).

This issue has been addressed on page 13, lines 396-399 and in the new Figure 6 reported below.

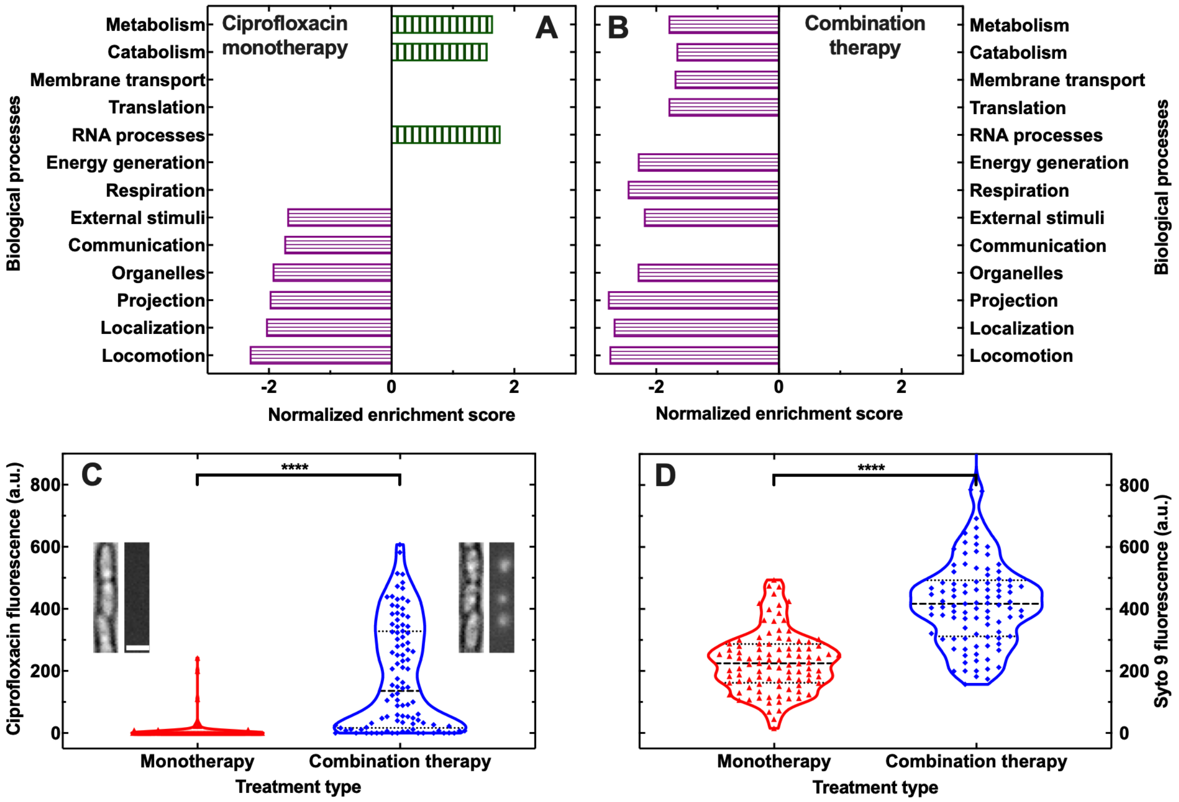

Figure 6 Synergistic bactericidal interactions between phage and ciprofloxacin are facilitated by phage-induced downregulation of membrane transport in *B. thailandensis*. Major biological processes that are significantly enriched and either up- or downregulated (vertically and horizontally patterned bars, respectively) in stationary phase *B. thailandensis* after 4 h of (A) monotherapy with ciprofloxacin at 0.125× MIC or (B) combination therapy with ciprofloxacin at 0.125× MIC and phage at an MOI of 1 with respect to untreated *B. thailandensis* incubated for 4 h in LB medium only. Corresponding differential gene expression analyses and gene ontology enrichment analyses are

reported in Supplementary files 1-3 and 4-6, respectively. If a process is not enriched there is no corresponding data bar. Distribution of fluorescence values of (C) ciprofloxacin-NBD or (D) Syto 9 accumulating in N = 100 individual stationary phase *B. thailandensis* that had been incubated in either ciprofloxacin-NBD or Syto 9 only or ciprofloxacin-NBD or Syto 9 and phage (red triangles and blue diamonds, respectively). Ciprofloxacin-NBD, Syto 9 and phage were introduced in the microfluidic device at t = 0 at a concentration of 32 μg ml^{-1}, 3.34 μM and 10^8 PFU ml^{-1}, respectively. Dashed lines indicate the median of each distribution, dotted lines indicate the quartiles of each distribution. **** indicate a p-value < 0.0001. Insets in (C): representative brightfield and fluorescence images of *B. thailandensis* after 4h of monotherapy (left) or combination therapy (right). Scale bar: 2 μm.

The authors state that the combination of phage and antibiotics could be advantageous in situations where it is difficult to achieve high antibiotic concentrations (in vivo, within biofilms), but do not provide data demonstrating improved sterilization of Burkholderia in animal models or in biofilm models.

Following the Reviewer's suggestion, we have now performed new experiments measuring and contrasting the effect of mono- and combination therapy in *Galleria mellonella* larvae, a simple infection model which has been increasingly used for microbiological research (Menard et al. Front. Cell. Infect. Microbiol. 10.3389/fcimb.2021.782733 2021). We found that ΦBp-AMP1 did not have any toxic effect on the larvae that survived exposure to phage alone as much as uninfected control larvae manipulated with PBS only; in contrast larvae treated with 10^4 *B. thailandensis* cells ml^{-1} rapidly died (Figure 5C). Phage monotherapy was able to significantly delay death (p-value < 0.0001). Crucially combination therapy with phage and finafloxacin allowed for significantly improved survival compared to finafloxacin monotherapy (p-value = 0.02 for combination vs finafloxacin monotherapy at 50 mg kg^{-1}, Figure 5D). Taken together these data show that the use of ΦBp-AMP1 increases antibiotic efficacy against stationary phase *B. thailandensis* both *in vitro* and in a simple *in vivo* infection model.

This issue has been addressed on page 11, lines 332-341 and in the new Figure 5 reported below, the corresponding new methods are described on page 22, lines 680-698.

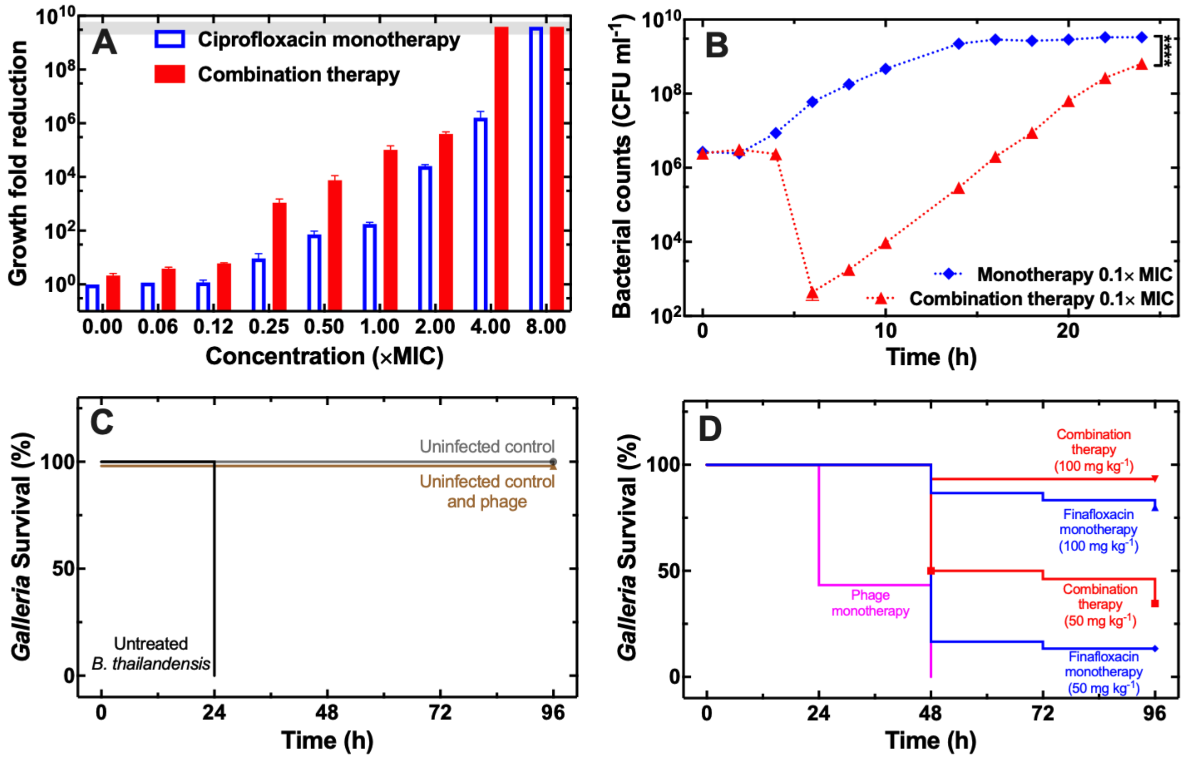

Figure 5 Synergistic bactericidal effect of phage and ciprofloxacin combination therapy. (A) Dependence of the ratio between the number of bacteria viable after 24 h incubation in LB medium and the number of bacteria viable 24 h ciprofloxacin monotherapy (blue bars) or phage-ciprofloxacin combination therapy (red bars) on the concentration of ciprofloxacin employed. In all cases the starting inoculum was stationary phase *B. thailandensis* at a concentration of 5×10^{5} CFU mL^{-1} and bacteria were counted at 24 h via CFU assays. The grey horizontal band represents a survival fold reduction that corresponds to the complete eradication of the bacterial population, i.e. colony counts below limit of detection of 10 CFU mL^{-1}. Pair-wise t-tests revealed that combination therapy yielded a significantly (p-value < 0.0001) higher growth reduction at all ciprofloxacin concentrations tested compared to ciprofloxacin monotherapy, apart from 8× MIC. (B) Temporal dependence of bacterial counts following 0.125× MIC ciprofloxacin monotherapy (blue diamonds) or combination therapy with 0.125× MIC ciprofloxacin and phage at an MOI of 1 (red triangles). Symbols and error bars are means and standard errors of the mean of biological triplicates each containing technical triplicates. Very small error bars cannot be visualised due to overlap with the datapoints. Dotted lines are guides-for-the-eye. Survival probability of *Galleria mellonella* larvae injected with (C) PBS only (uninfected control, grey circles and line), with PBS and 10^{5} phage ml^{-1} (uninfected control and phage, brown triangles and line), with 10^{4} *B. thailandensis* cells ml^{-1} (untreated *B. thailandensis*), or with (D) 10^{4} *B. thailandensis* cells ml^{-1} and either 10^{5} phage ml^{-1} (phage monotherapy, magenta circles and line), or 50 or 100 mg kg^{-1} of finafloxacin (combination monotherapies, red downward triangles or squares and lines) or 50 or 100 mg kg^{-1} of finafloxacin and 10^{5} phage ml^{-1} (blue upward triangles or squares and lines). Each treatment was carried out in biological triplicate, using 10 larvae in each replicate.

Minor issues

Reviewer 1

1. The study did an excellent job of examining different phage MOIs and antibiotic concentrations. In development of therapies that leverage phage-antibiotic synergy, sometimes there is consideration of the relative timing of administering these antimicrobials. In particular, they could be co-administered, versus a different strategy

involving either the phage or antimicrobial administered first and then 'chased' with the other at a later time point. Understandably, these types of manipulations fall outside the scope of the current study, and I am not asking that the authors conduct additional experiments. However, I would enjoy hearing their thoughts on the newly discovered biological mechanism in this study, and whether/how such differing strategies for administering the antimicrobials could matter.

We agree with the reviewer that a further factor often discussed to leverage phage-antibiotic synergy is the impact of sequential deployment of these antimicrobials. This approach has shown prior success, for example, against multidrug resistant *Pseudomonas aeruginosa* (Chan et al. Scientific Reports 10.1038/srep26717 2016). Considering that *B. thailandensis* downregulates efflux pumps during exposure to ΦBp-AMP1, we anticipate that phage administration followed by antibiotic administration could produce a stronger bactericidal effect than simultaneous administration. However, we recognise that further work will be required to investigate the potential benefit of staged administration.

This issue has been addressed on page 16, lines 498-504.

2. This study uses B. thailandensis strain E264 as a model, which can be highly useful because studies can leverage the prior literature on model strains to more straightforwardly understand empirical, genetic etc. results. Although I favor such approaches, as I read the paper I became curious whether strain E264 is closely versus distantly related to clinically relevant bacteria. More broadly, I wonder whether the observations in this study would likely benefit development of therapeutic strategies against other Burkholderia bacteria species. Again, I believe such studies fall outside of the current work, and there is some time spent in the Discussion nicely describing prior work in these other pathogen systems. Nevertheless, I felt the authors might want to place the work in context of likely versus unlikely immediate usefulness in building out development strategies targeting many different B. thailandensis strains and/or other Burkholderia species. I am not asking the authors to be overly speculative, but perhaps they can address this briefly in the Discussion.

The phage employed in this study, ΦBp-AMP1, was previously reported to effectively lyse different strains of both *B. thailandensis* and *B. pseudomallei* (Shan et al. Front. Microbiol. 10.3389/fmicb.2014.00599 2014; Gatedee et al. Virol. J. 10.1186/1743-422X-8-366 2011; Egilmez et al. Sci. Rep. 10.1038/s41598-018-27716-3 2018). Therefore, we expect that combination treatments using ΦBp-AMP1 will boost antibiotic accumulation and efficacy also against *B. pseudomallei*, the causative agent of melioidosis, although we recognise that further research is needed to optimise treatments against this closely related species.

This issue has been addressed on page 15, lines 467-471.

3. Related to the above comment, I felt the paper could better inform the reader of the health impact of Burkholderia bacteria, such as the patient communities particularly affected and/or the geographic regions that might be experiencing relatively high caseloads.

The authors included some nice references, but they could include a bit more information in the text of the Introduction.

We have now provided a brief account on the health impact of *Burkholderia*. *Burkholderia (B.) pseudomallei*, *B. thailandensis* and *B. mallei* (known as the Bptm group) cause life-threatening diseases. Of particular interest is *Burkholderia pseudomallei* which is endemic in at least 79 tropical and subtropical countries and causes the life-threatening disease melioidosis. Case numbers of melioidosis are closely linked to extreme weather events with heavy rainfall and environmental disturbance, such as landslides or construction work, exacerbating outbreaks. In northern Australia, First Nations people are often disproportionally impacted, likely due to comparatively higher numbers of people living in regions with endemic melioidosis, as well as often poor access to state-of-the-art treatment.

This issue has been addressed on page 3, lines 57-65.

Reviewer 2

1. line 45 – define "Bptm"

We have now clarified that taken together *Burkholderia (B.) pseudomallei, B. thailandensis* and *B. mallei* are known as the Bptm group.

This issue has been addressed on page 3, lines 57-58.

2. line 51 – "as well as changes in the global environment" – not sure what this means here

We have now clarified that environmental dispersion of antibiotics in ground water through livestock excretions and increased endemic bacterial spread due to global warming also contributes to the spread of acquired genetic resistance.

This issue has been addressed on page 3, lines 70-72.

3. line 59/61 – antibodies mentioned twice.

We have now removed the second mention of antibodies.

This issue has been addressed on page 3, line 81.

4. line 62 – "antimicrobial agents cannot change or adapt in real time" – this is also true of standard antimicrobials…and is it desirable for an antimicrobial agent to adapt in real time?

We have now clarified that these antimicrobial agents cannot evolve to overcome bacterial resistance, a limitation shared with antibiotic molecules routinely employed in healthcare (Torres-Barcelo and Hochberg Trends in Microbiology 10.1016/j.tim.2015.12.011 2016).

This issue has been addressed on page 3, lines 81-83.

5. line 70 – "seven" years or "several" years?

We have now changed from "seven" to "recent" years.

This issue has been addressed on page 4, lines 91.

6. line 77-78 – would be appropriate here to also discuss other ways in which phage/antibiotics synergize, e.g. downregulation of efflux systems that serve as phage receptors (as in work of Paul Turner and colleagues)

We have now added that the evolution of bacterial resistance to phage that use efflux pumps as receptors can alter the efflux pump mechanism, causing increased sensitivity to antibiotics (Chan et al. Scientific Reports 10.1038/srep26717 2016).

This issue has been addressed on page 4, lines 102-104.

7. line 143 – "bacterial micro-cultures" – not clear if this is somehow different from small scale cultures in 96 well plates (methods don't describe anything but this) – is it critical to have a certain inoculum size to observe the "zero" growth phenotype?

We have now clarified that indeed with bacterial micro-cultures we meant small-scale cultures in 96 well plates (each well containing 5×10^5 bacteria mL^{-1} in 100 µL LB as described in the methods). We have also clarified that in larger scale cultures (i.e. shaking flasks containing 2×10^6 bacteria mL^{-1} in 50 mL of LB) we did not observe such heterogeneity (Figure 5A) suggesting that it is critical to reduce the bacterial inoculum (from 10^8 to 10^5 bacteria) to capture a heterogeneous pool of mutants that are resistant to the phage as well as subpopulations that are susceptible to phage.

This issue has been addressed on page 6, lines 145-146 and 155-158.

8. line 171 – what does it mean to be a 'representative' high- or low-resistant survivor? What is meant by "unique" mutations – they were observed as the only genetic change present in the genome as compared to the parent?

We have now removed the word "representative" from the text since we understand that the Reviewer found it to be misleading. We have clarified that we have sequenced the genome of the five high-resistant and the five low-resistant survivor micro-cultures indicated with color circles and squares, respectively, in Figure 1C. We have also clarified that with "unique" mutations we meant the only genetic change observed when compared to the parental genome.

This issue has been addressed on page 7, lines 168-169, page 8, lines 180-181, and the revised Figure 1, reported below.

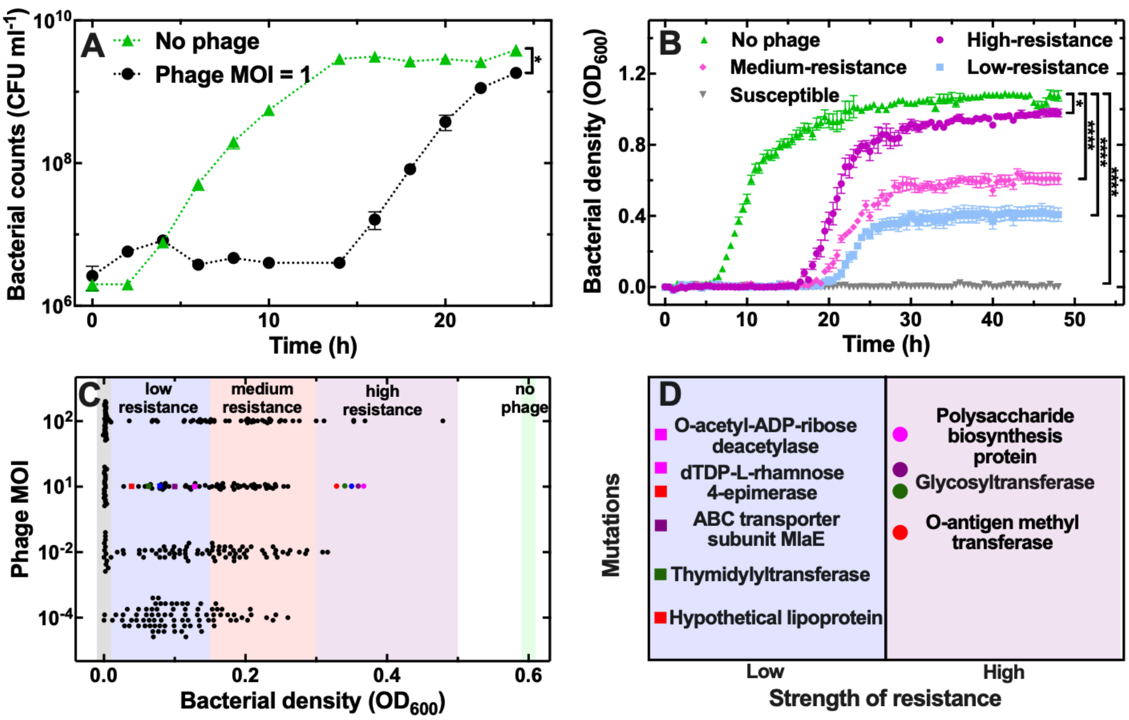

Figure 1 Heterogenous resistance to ΦBp-AMP1 in *B. thailandensis*. (A) Regrowth of stationary phase *B. thailandensis* populations in the presence of LB medium only (green triangles) or together with phage at an MOI of 1 (black circles). Symbols and error bars are means and standard errors of the means of CFU measurements obtained from biological triplicates each containing technical triplicates. Very small error bars cannot be visualised due to overlap with the datapoints. Dotted lines are guides-for-the-eye. Corresponding phage counts are reported in Figure S1. (B) Regrowth of stationary phase *B. thailandensis* populations in the presence of LB medium only (1.05 < OD_{600} < 1.2 after 48 h, green triangles) or together with phage at an MOI of 1 with different levels of bacterial resistance to phage emerging: high-resistance (0.9 < OD_{600} < 1.05 after 48 h, purple circles), medium-resistance (0.5 < OD_{600} < 0.7 after 48 h, magenta diamonds), low-resistance (0.3 < OD_{600} < 0.5 after 48 h, blue squares), susceptible (0 < OD_{600} < 0.01 after 48 h, grey downward triangles). Symbols and error bars are means and standard errors of bacterial density values, measured in OD_{600}, obtained from 84 technical replicates from biological triplicates. Very small error bars cannot be visualised due to overlap with the datapoints. * indicate a p-value < 0.05, **** indicate a p-value < 0.0001. (C). Bacterial density measurements after 24 h in the presence of phage at an MOI of 10^{-4}, 10^{-2}, 1 and 10^{2}. Each black circle represents a bacterial density value performed on one of 84 technical micro-culture replicates from biological triplicates. The coloured vertical bands in the background indicate the range of OD_{600} values for each level of resistance after 24 h: high-resistance (0.3 < OD_{600} < 0.5, purple band), medium-resistance (0.15 < OD_{600} < 0.3, magenta band), low-resistance (0.01 < OD_{600} < 0.15, blue band), susceptibility (0 < OD_{600} < 0.01, grey band). Coloured circle and square data points indicate populations that were whole genome sequenced; the corresponding unique mutations identified for each population are reported in (D).

9. line 312 – this should be noted as "data not shown"

We have now added this data to the new Figure S5 reported below.

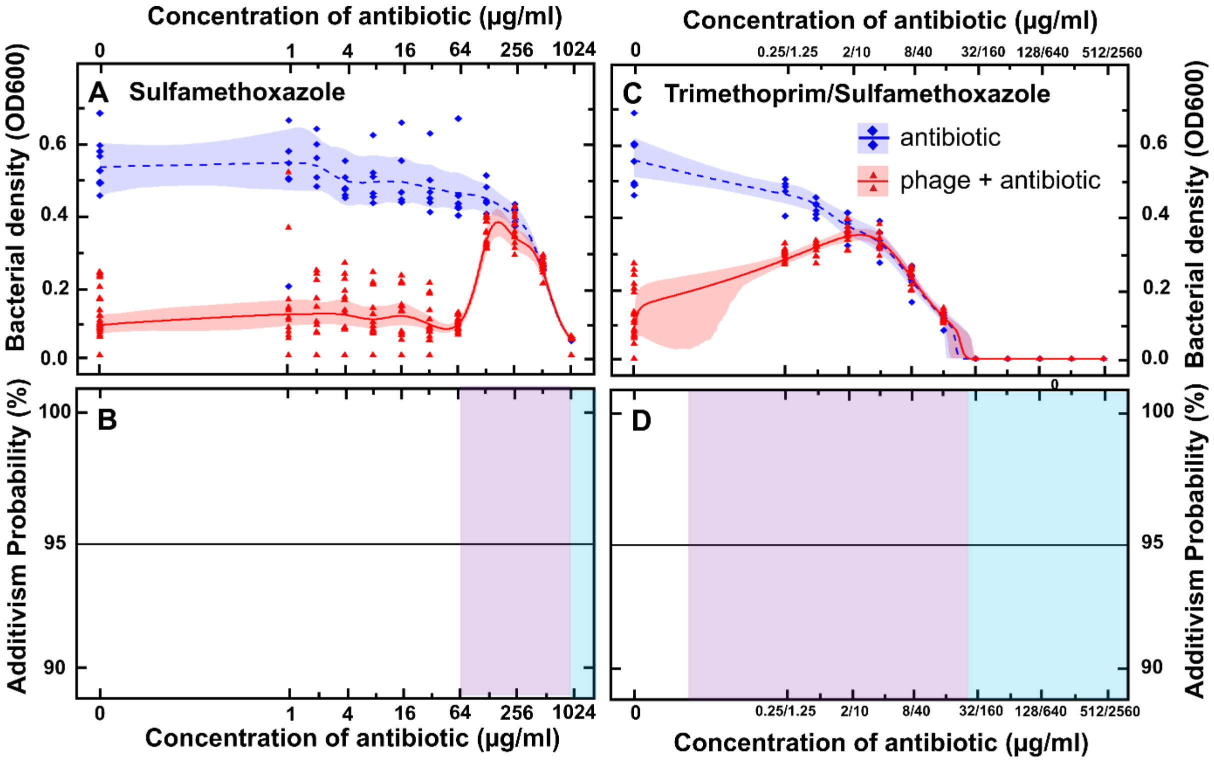

Figure S5 Interactions between ΦBp-AMP1 and folate synthesis inhibitors. Experimental data (symbols) and model predictions (lines and bands) describing the dependence of bacterial density on the concentration of (A) sulfamethoxazole and (C) trimethoprim/sulfamethoxazole (at a ratio of 1:5) in the absence (blue diamonds) and presence of phage ΦBp-AMP1 at an MOI of 1 (red triangles) after 24 h treatment. Each symbol represents the bacterial density measured in one of 15 technical replicates collated from biological triplicates. Some of the symbols overlap with each other. The lines and shaded areas are the medians, upper and lower quartiles, estimated by fitting our statistical non-linear regression model to our experimental data via Markov Chain Monte Carlo simulations. Corresponding predicted probability of an additive interaction between phage and (B) sulfamethoxazole and (D) trimethoprim/sulfamethoxazole (ratio 1:5) is below 90%. Not shaded or blue shaded areas indicate antibiotic concentration ranges where the phage or the antibiotic dominate, respectively. Purple shaded areas indicate antagonism.

10. line 380 – authors note the emergence of a phage-resistant subpopulation that grows to high density; what implications does this have for using phage plus antibiotics in clinical practice?

We have added a note that the 5-log reduction in bacterial population after 6h of combination therapy compared to antibiotic monotherapy might provide significant release for the immune system to be able to clear bacterial infections before the emergence of a phage-resistant subpopulation, although further research will be needed to test this hypothesis.

This issue has been addressed on page 11, lines 328-331.

11. line 452 – cipro-NBD is reportedly used at 32 ug/ml – what is the MIC for this modified cipro molecule? Are the bacteria viable in the combo phage/antibiotic condition?

We have now clarified that using the broth microdilution approach, we determined that the MIC of ciprofloxacin-NBD against stationary phase *B. thailandensis* E264 is 64 μg ml^{-1}. Therefore, we chose to deploy ciprofloxacin-NBD at 32 μg ml^{-1}, i.e. 0.5× MIC which is within the additive concentration range reported in Figure 3AB.

This issue has been addressed on page 13, lines 389-392.

12. line 472 (and abstract) – phage-antibiotic interactions are "underpinned by phage-induced downregulation…". Underpinned is an imprecise word – the relationship between the phenotype and the proposed mechanism, however, is not established.

We have now changed the word "underpin" to "facilitate", "support" or "enable" throughout the manuscript, depending on the context.

13. Fig 6 – what are the insets in panel C representing?

We have now clarified in the caption of Figure 6 that the insets are representative brightfield and fluorescence images of *B. thailandensis* after 4h of monotherapy (left) or combination therapy (right).

14. line 538 – "The outcome of phage-antibiotic therapy is often contradictory" – rephrase, this is not clear in its meaning.

We have now removed this sentence because we understand it was misleading and unnecessary in that context.

Reviewer 3

L163-167 Was there variation in the growth of 'survivor populations' between 'high' and 'low' resistance in this follow-up assay in liquid media (i.e., OD) and/or in colony size? L170-179. This is a relatively small sample size to infer how strength of resistance corelates to specific resistance mutations, especially considering that all mutations appear unique. Do you have data on the phage resistance of these individual sequenced clones, rather than their parent populations? E.g., growth in the presence/absence of phages, colony size to infer fitness costs.

Following the Reviewer's suggestion, we have taken the same five survivor low-resistance and five survivor high-resistance populations that we had genome sequenced (now indicated with colored squares and circles, respectively, in Figure 1C and 1D) and re-inoculated them into 96-well plates in the presence of phage for 48 h. We found that the high-resistance populations started growth earlier and reached higher final densities compared to low-resistance populations (Figure S4A and S4B, respectively). As expected, we found a positive correlation between the final optical density at the end of this second 48h exposure to phage and the optical density measured for each survivor population at the end of the first 48h exposure to phage ($r = 0.81$, **, Figure S4C) and a negative correlation between the onset of growth during this second exposure to phage and the optical density measured for each survivor population at the end of the first 48h exposure to phage ($r = -0.69$, *, Figure S4D).

This issue has been addressed on page 7, lines 168-177 and in the new Figure S4, reported below.

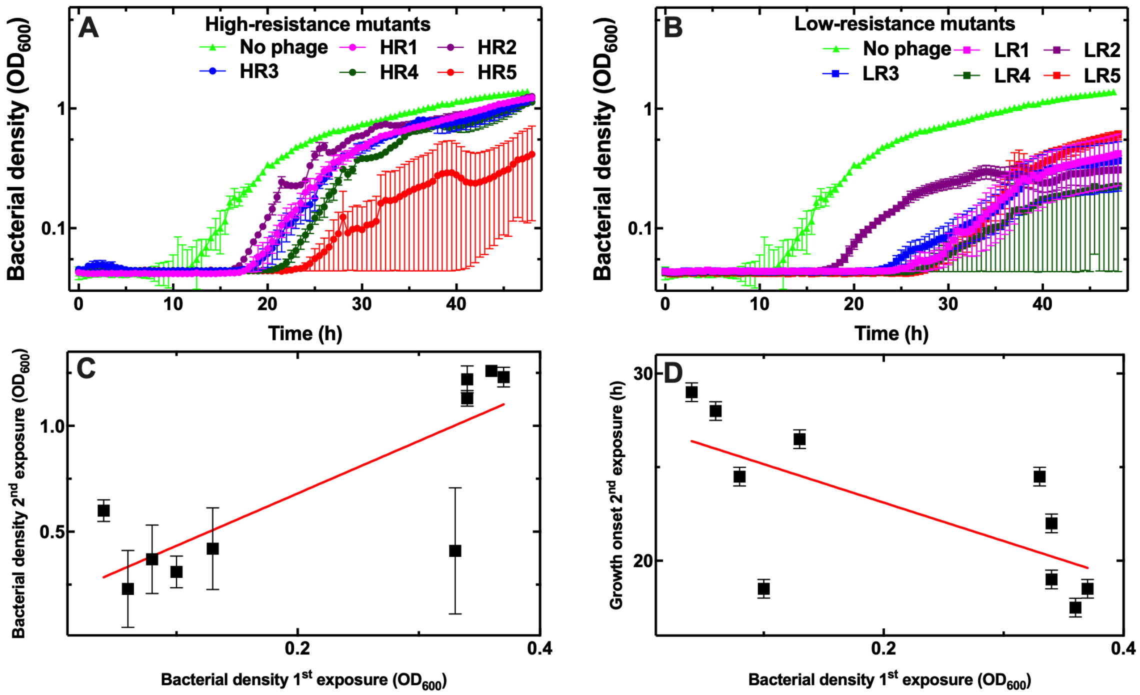

Figure S4 Regrowth of resistant mutants in the presence of phage. Growth of (A) high-resistant and (B) low-resistant mutants when re-inoculated in 96-well plates in the presence of phage at an MOI of 1 for 48h. Data points for each mutant have been color coded as in Figure 1C and 1D, HR and LR stands for high-resistant and low-resistant, respectively, and mutants have been numbered in descending order of resistance according to the data in Figure 1C, i.e. HR1 is the highest resistant mutant whereas LR5 is the lowest resistant mutant investigated. Green triangles represent the growth of the parental strain in the absence of phage. Symbols and error bars are means and standard errors of bacterial density values, measured in OD_{600}, obtained from 9 technical replicates from biological triplicates. Corresponding correlation between (C) the final optical density at the end of the second 48h exposure to phage and the optical density measured for each survivor population at the end of the first 48h exposure to phage and (D) the onset of growth during the second exposure to phage and the optical density measured for each survivor population at the end of the first 48h exposure to phage. The red lines are linear regressions to the data.

L356. End of sentence missing?

Apologies, the end of the sentence was hidden behind Figure 5, we have now fixed this issue on page 11, lines 314.

L435. Would be interesting to see these specific examples included in Figure 6 to help compare the degree of enrichment/downregulation in context to other affected functions.

Following the Reviewer's suggestion, we have now produced a new Figure S8, reproduced below, reporting the differential expression of the genes discussed on page 13, lines 373-378.

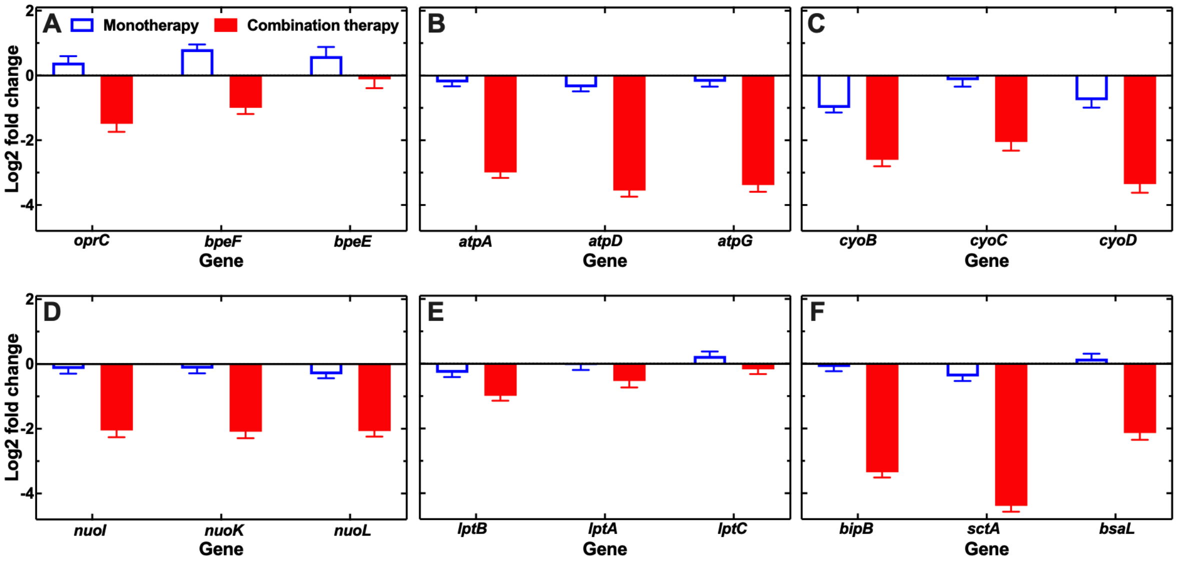

Figure S8 Differential expression of genes involved in efflux, ATP generation, respiration, LPS transport and secretion during monotherapy or combination therapy with respect to untreated *B. thailandensis*. Downregulated (negative Log2-fold change) or upregulated (positive Log2-fold change) genes of interest in stationary phase *B. thailandensis* after 4 h of monotherapy with ciprofloxacin at 0.125× MIC (blue empty bars) or combination therapy with ciprofloxacin at 0.125× MIC and phage at an MOI of 1 (red filled bars) with respect to untreated *B. thailandensis* incubated for 4 h in LB medium only. Corresponding numerical values are reported in Supplementary files 1 and 3.

Figure 6A. I found it confusing to have different y-axis labels for plots A and B when they are aligned. It would be clearer to include all categories in both plots to enable a more informed comparison.

We have now modified Figure 6, reported below, to take into account the Reviewer's suggestion.

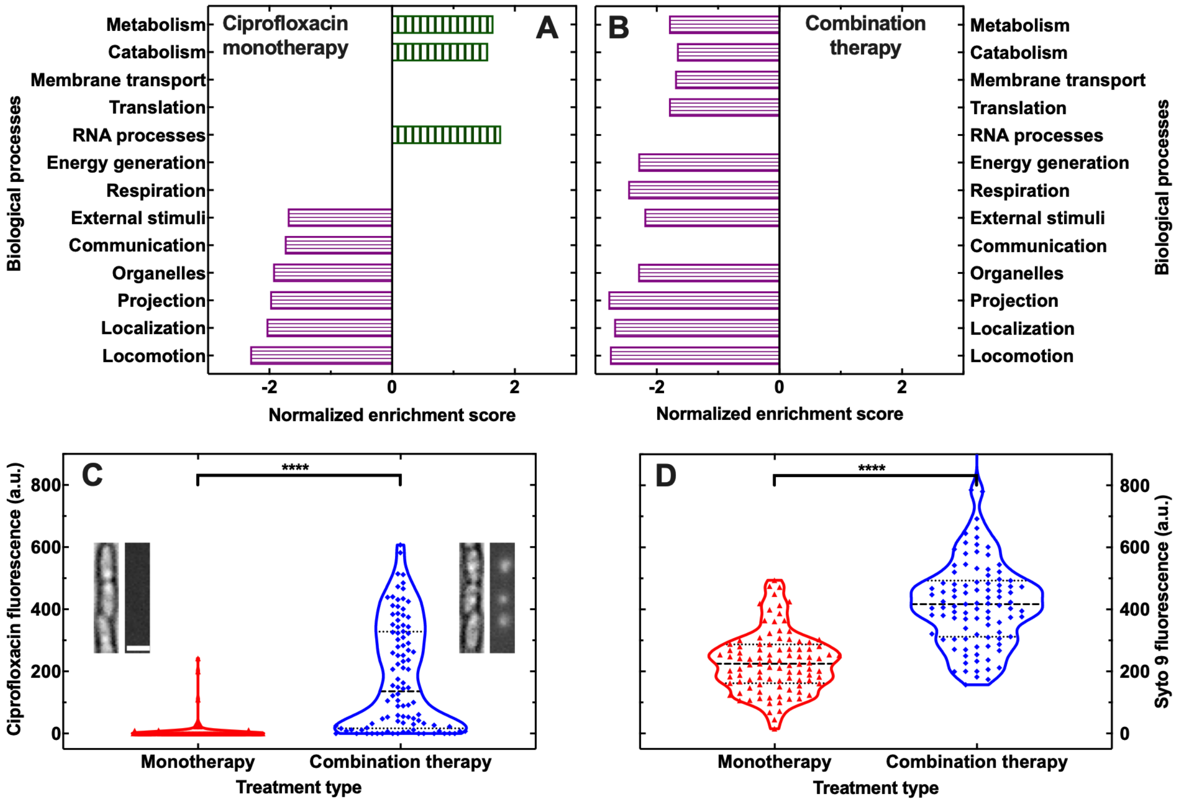

Figure 6 Synergistic bactericidal interactions between phage and ciprofloxacin are <u>facilitated</u> by phage-induced downregulation of membrane transport in *B. thailandensis*. Major biological processes that are significantly enriched and either up- or downregulated (vertically and horizontally patterned bars, respectively) in stationary phase *B. thailandensis* after 4 h of (A) monotherapy with ciprofloxacin at 0.125× MIC or (B) combination therapy with ciprofloxacin at 0.125× MIC and phage at an MOI of 1 with respect to untreated *B. thailandensis* incubated for 4 h in LB medium only. Corresponding differential gene expression analyses and gene ontology enrichment analyses are reported in Supplementary files 1-3 and 4-6, respectively. If a process is not enriched there is no corresponding data bar. Distribution of values of fluorescence of (C) ciprofloxacin-NBD or (D) Syto 9 accumulating in N = 100 individual stationary phase *B. thailandensis* that had been incubated in either ciprofloxacin-NBD or Syto 9 only or ciprofloxacin-NBD or Syto 9 and phage (red triangles and blue diamonds, respectively). Ciprofloxacin-NBD, Syto 9 and phage were introduced in the microfluidic device at t = 0 at a concentration of 32 μg ml^{-1}, 3.34 μM and 10^8 PFU ml^{-1}, respectively. Dashed lines indicate the median of each distribution, dotted lines indicate the quartiles of each distribution. **** indicate a p-value < 0.0001. Insets in (C): representative brightfield and fluorescence images of *B. thailandensis* after 4h of monotherapy (left) or combination therapy (right). Scale bar: 2 μm.
