# Supplementary material for: Phage-induced efflux down-regulation boosts antibiotic efficacy
Source: PLoS Pathog. 2024 Jun 28;20(6):e1012361. doi: 10.1371/journal.ppat.1012361 (PMC11239113; doi:10.1371/journal.ppat.1012361)
Supplement: Attachment — Submitted filename: PLoS Pathogens Kraus et al_Response to reviewers.pdf [file ppat.1012361.s033.pdf]

Major issue, Reviewer 2

The authors have responded to reviewer comments with revised text and new figures/data. I appreciate their responses, but would continue to advise that they temper their conclusions regarding the mechanism underlying phage-antibiotic synergy as being increased antibiotic efflux. The cipro-NBD and Syto9 experiments that they show do not distinguish between increased permeabilization of the small molecule versus efflux (both effects can contribute to increased fluorescence given the experimental design) - and since their transcriptomic data document decreased expression of LPS transport genes as well as BpeEF-OprC this caveat should be acknowledged.

Following the Reviewer's suggestion, we have now acknowledged that reduced antibiotic accumulation in the presence of phage could also be due to decreased expression of LPS transport genes in the presence of phage.

This issue has been addressed on lines 384-385, 421 and 540-543 of the revised manuscript.
